# Artificial Intelligence Technologies in the Microsurgical Operating Room (Review)

**DOI:** 10.17691/stm2023.15.2.08

**Published:** 2023-03-29

**Authors:** A.E. Bykanov, G.V. Danilov, V.V. Kostumov, O.G. Pilipenko, B.M. Nutfullin, O.A. Rastvorova, D.I. Pitskhelauri

**Affiliations:** Neurosurgeon, 7^th^ Department of Neurosurgery, Researcher; National Medical Research Center for Neurosurgery named after Academician N.N. Burdenko, Ministry of Healthcare of the Russian Federation, 16, 4^th^ Tverskaya-Yamskaya St., Moscow, 125047, Russia;; Academic Secretary; National Medical Research Center for Neurosurgery named after Academician N.N. Burdenko, Ministry of Healthcare of the Russian Federation, 16, 4^th^ Tverskaya-Yamskaya St., Moscow, 125047, Russia;; PhD Student, Programmer, the CMC Faculty; Lomonosov Moscow State University, 1 Leninskiye Gory, Moscow, 119991, Russia; PhD Student, Programmer, the CMC Faculty; Lomonosov Moscow State University, 1 Leninskiye Gory, Moscow, 119991, Russia; PhD Student, Programmer, the CMC Faculty; Lomonosov Moscow State University, 1 Leninskiye Gory, Moscow, 119991, Russia; Resident, 7^th^ Department of Neurosurgery; National Medical Research Center for Neurosurgery named after Academician N.N. Burdenko, Ministry of Healthcare of the Russian Federation, 16, 4^th^ Tverskaya-Yamskaya St., Moscow, 125047, Russia;; Professor, Head of the 7^th^ Department of Neurosurgery; National Medical Research Center for Neurosurgery named after Academician N.N. Burdenko, Ministry of Healthcare of the Russian Federation, 16, 4^th^ Tverskaya-Yamskaya St., Moscow, 125047, Russia;

**Keywords:** artificial intelligence, microsurgery, neural networks, microsurgical skills, machine learning

## Abstract

Surgery performed by a novice neurosurgeon under constant supervision of a senior surgeon with the experience of thousands of operations, able to handle any intraoperative complications and predict them in advance, and never getting tired, is currently an elusive dream, but can become a reality with the development of artificial intelligence methods.

This paper has presented a review of the literature on the use of artificial intelligence technologies in the microsurgical operating room. Searching for sources was carried out in the PubMed text database of medical and biological publications. The key words used were “surgical procedures”, “dexterity”, “microsurgery” AND “artificial intelligence” OR “machine learning” OR “neural networks”. Articles in English and Russian were considered with no limitation to publication date. The main directions of research on the use of artificial intelligence technologies in the microsurgical operating room have been highlighted.

Despite the fact that in recent years machine learning has been increasingly introduced into the medical field, a small number of studies related to the problem of interest have been published, and their results have not proved to be of practical use yet. However, the social significance of this direction is an important argument for its development.

## Introduction

In recent decades, there has been significant interest in the practical application of artificial intelligence (AI), including machine learning, in the field of clinical medicine. The current advances in AI technologies in neuroimaging open up new perspectives in the development of non-invasive and personalized diagnostics. Thus, methods of radiomics, i.e., extracting a large number of features from medical images, are actively developing. These features may contain information to describe tumors and brain structures which are not visible to the naked eye [1-5]. It is assumed that the correct presentation and analysis of images with neuroimaging features will help to distinguish between types of tumors and correlate them with the clinical manifestations of the disease, prognosis, and the most effective treatment.

Technologies that evaluate the relationship between features of tumor imaging and gene expression are called radiogenomics [6-9]. These methods are aimed at creating imaging biomarkers that can identify the genetic signs of disease without biopsy.

The AI advances in the analysis of molecular and genetic data, signals from invasive sensors, and medical texts have become known as well. The universality of approaches to the use of AI opens up new, original ways of using them in the clinic.

From a technical point of view, the term “artificial intelligence” can denote a mathematical technology that automates the solution to some intellectual problem traditionally solved by a person. In a broader sense, this term refers to the field of computer science in which such solutions are developed.

Modern AI relies on machine learning technologies — methods for extracting patterns and rules from the data representative of a specific task (medical images, text records, genetic sequences, laboratory tests, etc.). For example, AI can find “rules” for predicting poor treatment outcomes from a set of predictors by “studying” retrospectively a sufficient number of similar cases with known outcomes. This AI property can be used in solving tasks of automating individual diagnostic processes, selecting treatment tactics, or predicting outcomes of medical care according to clinical findings.

In medical practice, particularly in surgery, AI, along with surgical robots, 3D printing and new imaging methods, provides solving a wide range of problems, increasing the level of accuracy and efficiency of operations.

The use of AI is even more important in microsurgery, when it comes to interventions on small anatomical sites with the use of optical devices and microsurgical tools.

An AI challenge in microsurgery is the automatic recognition of anatomical structures that are critical for the microsurgeon (arteries, veins, nerves, etc.) in intraoperative photographs, video images, or images of anatomical preparations. The solution to this problem creates prospects for the development of AI automatic alert tools at the risk of traumatization of critical structures during surgery in real time, the choice of trajectories for safe dissection or incisions in functionally significant areas [10].

Artificial intelligence can evaluate handling of surgical instruments, check the positioning of the micro instrument in the surgeon’s hands (its position in the hand, position to the surgical wound), and hand tremor during surgery.

Determining a phase of surgery, predicting outcomes and complications, and creating the basis for an intelligent intraoperative decision support system are prospective goals for AI in microsurgery.

A non-trivial task of using AI in microsurgery is to assess the skills of novice surgeons and residents, as well as improve the skills of more experienced specialists. The solution to this problem, due to the extreme work complexity and responsibility of a microsurgeon, will bring this field of medicine to new frontiers.

To assess the available solutions to the issue of using AI in the microsurgical operating room, an analysis of articles in the PubMed text database of medical and biological publications was performed. Literature search was carried out using the key words “surgical procedures”, “dexterity”, “microsurgery” AND “artificial intelligence” OR “machine learning” OR “neural networks” among articles in English and Russian with no limitation to publication date.

## Automatic assessment of the level of microsurgical skills

Continuous training and constant improvement of microsurgical techniques are essential conditions for the formation of a skilled microsurgeon. It often takes most of the professional life to acquire the required level of microsurgical skills [11-13].

Microsurgical training requires constant participation of a tutor who would correct non-optimal actions and movements of the microsurgeon and supervise the learning process. A parallel could be drawn between the training of microsurgeons and Olympic athletes: achieving a high level is impossible without a proper training system and highly qualified coaches. However, due to the high clinical workload and strenuous schedule of skilled microsurgeons-tutors, their permanent presence in the microsurgical laboratory is impossible, and the start of training in a real operating room is in conflict with the norms of medical ethics. In this situation, AI technologies can be used in the learning process to control the correctness and effectiveness of the manual actions of a novice neurosurgeon.

To date, the set of AI technologies that would be adapted for the analysis of microsurgical manipulations is significantly limited. For example, the use of accelerometers attached to microsurgical instruments to assess the level of microsurgical tremor was described in the papers by Bykanov et al. [14] and Coulson et al. [15]. In the work by Harada et al. [16], infrared optical motion tracking markers, an inertial measurement unit, and load cells were mounted on microsurgical tweezers to measure the spatial parameters associated with instrument manipulation. AI and machine learning methods were not applied in this work. Applebaum et al. [17] compared parameters such as the time and number of movements in the process of performing a microsurgical task by plastic surgeons with different levels of experience, using an electromagnetic motion tracking device to record the movement of the surgeon’s hands. This approach to the assessment of microneurosurgical performance stands out for its objectivity and reliability of instrumental measurements, but requires special equipment.

Expert analysis of video images of the surgeon’s work in the operating room is an alternative method for assessing the degree of mastering microsurgical techniques. However, involving an expert assessor in the analysis of such images is a time-consuming and extremely laborious method. Frame-by-frame analysis of microinstrument motion based on video recordings of a simulated surgical performance was applied by Óvári et al. [18]. Attempts to objectively evaluate and categorize the microsurgical effect based on the analysis of a video recording of a microsurgical training were made by Satterwhite et al. [19]. However, the analysis and evaluation of the performance of trained microsurgeons in this work were carried out by the expert assessors by viewing video recordings and grading according to the developed scale, which does not allow leveling the influence of the subjective factor on the results of the analysis.

A promising alternative to these technologies is machine learning methods, computer vision, primarily, for automated evaluation of the effectiveness of macro- and microsurgical performance. These methods can be applied on the base of the detection and analysis of microsurgical instrument motion in the surgical wound. After analyzing the limited scientific literature on this topic, we summarized the main processes for obtaining data for the analysis of microsurgical procedures using machine learning (Table 1).

**Table 1:** Features of data mining process used in machine learning, for the analysis of microsurgical manipulations

Data mining process	Description	Advantages	Disadvantages
Recording of interoperative features	Recording interoperative features such as intra-abdominal pressure, weight of suction and irrigation bags, surgical table tilting, etc.	1. Easy recording, no additional equipment is required in the operating room	1. Frequent manual feature recording 2. Time-consuming process
Manual annotation of using instruments	Manual annotation of points in time when each instrument is entered or withdrawn from use	1. High accuracy 2. Strong correlation with the main surgical working process 3. No additional equipment is needed in the operating room	1. Time-consuming process
Using labeled instruments	Tool use detection by affixing radiofrequency identification tags to each instrument and placement of antennas all over the operating room. The antennas detect the instrument as “activated” when the surgeon picks it up	1. Avoiding laborious manual annotation 2. Strong correlation with the main surgical working process	1. The process needs special additional equipment in the operating room
Video-based automatic tool usage detection	Automatic tool usage detection in the surgery video using machine learning models	1. Avoiding laborious manual annotation and no need for additional equipment in the operating room 2. Strong correlation with the main surgical working process	1. Small loss of accuracy compared to manual annotation
Manual feature extraction from video	It involves manual identification of various types of features from video images, such as texture, color histograms, object shape detection	1. Taking into account additional features to determine the phases of operations 2. No additional equipment in the operating room is required	1. Features are created manually and determined in advance, which means that information useful to machine learning algorithms can be lost
Automatic feature extraction from video	Some models are able to automatically learn and identify important features of surgical procedures using video images	1. The learned features can provide the most discriminatory power for phase recognition as they take into account all the data	1. Training may be technically difficult, labor-consuming and require significant computational resources

The few scientific literature data indicate that machine learning methods allow to identify complex relationships in the movement patterns of a microsurgeon and predict the parameters of the effectiveness of microsurgical performance. To implement these tasks, the first step is to train the model to correctly classify the motion and the microsurgical instrument itself in the surgery video. The ongoing research studies in this direction are mostly focused on teaching computers two main functions: determining the phase of a surgical operation and identifying a surgical instrument [20].

In works on microsurgery using machine learning, two types of data sources are most often used: these are video recordings of surgery [21] and a set of variables that are obtained from sensors attached to microinstruments or on the body of the operating surgeon. Some studies combine both sources [22].

In the study by Markarian et al. [21], the RetinaNet, a deep learning model was created for the identification, localization, and annotation of surgical instruments based on intraoperative video recordings of endoscopic endonasal operations. According to the findings of the study, the developed model was able to successfully identify and correctly classify surgical instruments. However, all the instruments in the work belonged to the same class — “instruments”.

An interesting study was carried out by Pangal et al. [23]. In this work, the authors evaluated the ability of a deep neural network (DNN) to predict blood loss and damage to the internal carotid artery based on the 1-minute video data obtained from a validated neurosurgical simulator for endonasal neurosurgery. The prediction results of the model and expert assessors coincided in the vast majority of cases.

In the work by McGoldrick et al. [24], researchers used video recordings made directly from the camera of the operating microscope and the ProAnalyst software to analyze the smoothness of movements of a vascular microsurgeon performing microanastamosis, using a logistic regression model and a cubic spline.

Franco-González et al. [25] designed a stereoscopic system with two cameras that recorded images from different angles of surgical tweezers. The 3D motion tracking software was created using the C++ programming language and the OpenCV 3.4.11 library.

Oliveira et al. [26] showed in their work that the use of machine learning and computer vision in the simulation of microsurgical operations provides enhancing basic skills of both residents and experts with extensive experience.

The use of neural networks with long short-term memory (LSTM) in the analysis algorithms became a major advance in the process of surgical phase recognition, which made it possible to improve the accuracy of determining a surgical phase up to 85–90%.

It is important to note that due to typical data volume limitations, model developers often use the so-called transfer learning [27], which allows the model to be pre-trained on the same data (most often, on open sets that solve similar problems in the same subject area) and then retrain on others, on which the target problem is solved. Currently, the following sets of open data are known, which are used in solving problems related to assessing the accuracy of surgical operations:

EndoVis Challenge datasets are a collection of labeled datasets that contain videos of various types of surgical operations for classification, segmentation, detection, localization, etc. [28];

Cholec80 contains 80 videos of endoscopic operations performed by 13 different surgeons; all videos are labeled taking into account the phases of operations and the presence of instruments in the frame [29];

MICCAI challenge datasets are ones that allow a large number of contests in the analysis of medical data, including the analysis of surgical materials [30];

JHU-ISI and JIGSAWS, a labeled dataset of video recordings of operations performed by eight surgeons having three skill levels who performed a total of 103 basic robotic laboratory tests [31];

ATLAS Dione have 99 videos of 6 types of surgeries performed by 10 different surgeons using the da Vinci Surgical System. The frame size is 854×480 pixels, each of which is labeled for the presence of surgical instruments in the frame [32].

Theoretically, hundreds and thousands of videos can be used to analyze them using machine learning methods. However, to train the model, it is necessary to view and perform video image labeling in the “manual mode”, which requires a lot of time. A possible solution to this problem is the use of new algorithms that provide annotating video files independently [33].

Table 2 shows a list of machine learning methods used, according to scientific literature, in the analysis of video images of microsurgical interventions, with a brief description of them.

**Table 2:** Machine learning methods used in the analysis of data from microsurgical interventions

Algorithm	Description	Advantages	Disadvantages
Hidden Markov model (HMM) [34, 35]	Statistical model based on Markov processes. A probabilistic approach modeling a set of observable/hidden states and the probability of transition between hidden states. Having detected the transition of observed states (for example, bimanual motion of the instrument), the algorithm evaluates the most probable sequence of hidden states (for example, in the task of suturing). Hidden states often represent surgical maneuvers, and metrics can be derived from hidden state transitions. The data obtained can be used to analyze the surgeon’s performance	1. Low model complexity 2. Relatively less training data is required 3. The algorithm is efficient when modeling temporal information	1. Gesture segmentation from motion data may be challenging 2. Parameter setting and model development can be time-consuming 3. The functions used in the model are defined manually
Dynamic time warping algorithm (DTW) [36, 37]	An algorithm that finds the best match between two time sequences that differ in time or speed	1. Ease and convenience in implementation 2. Highly effective in the search for similarities/correspondences between two sequences	1. Functions must be defined manually 2. Only two sequences can be compared simultaneously 3. Long computation time when searching for the optimal match
Support vector machines algorithm (SVM) [38, 39]	A method for creating a delimiting linear hyperplane based on the geometric distance between data. Designed for supervised machine learning that learns the hyperplane or decision boundary between classes. The hyperplane is derived by maximizing the geometric distance between class support vectors. New data will be projected into hyperspace and subsequently classified based on the ratio to the hyperplane	1. Non-linear classification is possible by applying a kernel 2. Can be adapted for regression 3. Easy to understand with low common error 4. Low computational complexity of inference	1. Difficult to implement for large training data 2. Difficult to solve problems with multiple classifications 3. Sensitive to missing data, parameters and choice of kernel functions
k-nearest neighbors algorithm (kNN) [40]	A supervised learning algorithm (by precedents) for classification, in which a new point is classified by k-nearest neighbors from the training set. The algorithm groups the points of each class together. During inference, the Euclidean distances between the new observed data point and the training data points are calculated. Then, k-nearest neighbors (i.e. the k-points with the shortest distances to the observed point), and the new data point will be labeled as the class with the most number in k-nearest neighbors	1. No training in a classic way is required 2. Low complexity of the algorithm 3. Suitable for multi-class classification tasks 4. Low “cost” of retraining 5. Improved handling of overlapping data fields	1. Poor performance when using high-dimensional data 2. “Lazy” learning, long inference time with large datasets 3. Sensitive to noise, missing data, and outliers 4. Data scaling function is required 5. Poor performance with class-unbalanced datasets
Naive Bayes algorithm [41]	Supervised machine learning algorithm for classification based on Bayes’ theorem. A simplified version of the Bayes algorithm is a naive Bayes approach built with the assumption that the features are conditionally independent. Class with the highest posterior probability is the result of the prediction	1. Simple, reliable, and easy to interpret logic 2. Insensitive to missing data 3. Works well when features are close to conditionally independent ones 4. Works well with small datasets	1. Hypothesis of conditional independence is required 2. Tends to perform worse the more complex models with large datasets or correlated features are used 3. Prior probability is required
Decision trees [40]	Supervised learning algorithm for classification. The data are repeatedly split into subsets and eventually classified at the end nodes according to the logic of the nodes along the way	1. Simple and easy to interpret algorithm 2. Suitable for big data 3. Low computational power 4. Requires no domain knowledge or parameter assumptions 5. Not susceptible to loss of function 6. Human logics-based and deterministic	1. Prone to retraining 2. Can be unstable as small data changes can lead to a new tree architecture 3. Calculations can get very complex 4. Time sequences are difficult to classify 5. Pre-processing and feature selection are required 6. Sequential process, it cannot be parallelized
Random forest [42]	Supervised learning algorithm for classification based on decision trees. The algorithm combines several randomly generated decision trees	1. Reduces the need for retraining in the decision tree and improves accuracy 2. Flexibility to regression problems 3. Resilience to missing data 4. High learning rate	1. Considerable computational power may be required 2. Likely to be unstable since small changes in data can lead to new tree architecture 3. Difficult or hardly possible to interpret in some nodes of features 4. High computational cost in inference with multiple sequential processes
Logistic regression [43]	Supervised learning algorithm for classification based on logistic (or sigmoid) function	1. Ease of understanding, interpretation, and implementation 2. High performance 3. Good accuracy for very simple datasets	1. Can be easily outperformed by more complex algorithms 2. Difficulties in solving non-linear problems 3. Sensitive to blurry features
Principal component analysis (PCA) [44]	Unsupervised learning algorithm for data dimensionality reduction. Fits the data linearly along the eigenvectors (with the largest eigenvalues). As a result, the directions with the highest dispersion are selected	1. Improvement of data visualization 2. Improvement of algorithm performance 3. Correlated features are removed	1. Principal components (linear combinations of original features) are abstracted information from the data and can be difficult to interpret 2. Sensitive to the scale of features and outliers 3. Compromise between information loss and dimensionality reduction
Linear discriminant analysis [40]	Supervised learning method for dimensionality reduction and classification. A statistical method that projects data onto new axes which maximize separability between classes by maximizing interclass variance and minimizing intraclass variance	1. Controlled dimensional reduction with prior knowledge of classes 2. Can outperform principal component analysis as a dimensionality reduction method	1. Not suitable for non-Gaussian samples 2. Prone to overfitting 3. The projection space cannot exceed the existing dimensions 4. Limited by sample type
Clustering by k-means [45]	An iterative clustering algorithm (unsupervised) that separates unlabeled data into k distinct groups. Therefore, observations having similar features are grouped together. The decision for a new point to be grouped into one of the k-groups is based on its minimum distance from the center of the group. Centers will be recalculated iteratively until convergence. Then, the means of the clusters will be used to determine the classes of new observed data points	1. Ease of implementation 2. Low complexity of the algorithm 3. Scaling to large datasets	1. Necessity to assign k that do not meet some classification requirements 2. Sensitive to outliers and initial values 3. Difficulty in clustering data of different sizes 4. Difficulty for implementation in the case of high-dimensional data 5. Not suitable for “non-convex” classification
ANN and DNN (artificial and deep neural networks) [46]	A collection of artificial neurons that interact with each other. An ANN is a network of nodes (or neurons) connected to each other to represent data or approximations. DNN is an ANN with many layers (i.e. deep layers). Deep ANNs can learn and determine optimal features from data that can be generalized to get the best classification results under implicit scenarios	1. The algorithm can achieve high accuracy 2. Ability to model complex and non-linear problems 3. Ability to learn patterns and generalize to process unseen data 4. Reliable and fault-tolerant to noise	1. A large amount of training data is required 2. Time-consuming learning process and need for significant computational power to train complex networks 3. Difficulty in interpretation due to its “black box” 4. The learning process is stochastic — even learning with the same data can lead to receiving different networks
Convolutional neural networks (CNN) [47]	CNN is an artificial neural network with a “deep” structure, as well as layers of convolution operations and pooling layers. The CNN has the ability to learn the best representation of features which are then used for a statistically shift-invariant classification of input information based on its hierarchical structure	1. Learning representative features from data 2. Handling data with noise and lack of information 3. Wide use for high-resolution image classification 4. Pooling can abstract high-level information 5. Learning can be parallelized	1. Time-consuming learning process and there is need for significant computational power (compared to common methods of machine learning) 2. The pooling function results in the loss of detailed and valuable information 3. Poor performance at low resolution of an input image
Recurrent neural networks (RNN) [46]	RNN networks are a kind of neural network architecture, where connections between elements form a directed sequence. They are designed for modeling sequential processes. They use the current observation together with the output of the network in the previous state to generate the output	1. Parameter sharing mechanism, Turing completeness 2. The ability to memorize makes the algorithm suitable for processing time series signals, including semantic analysis of text, classification of its emotional coloring, and language translation	1. Difficult to train 2. Imperceptible problem with vanishing gradient 3. Gradient explosion problem which can be solved using clipping gradient 4. Problems with short-term memory
Long short- term memory networks (LSTM) [48]	An LSTM network is an artificial neural network containing LSTM modules instead of or in addition to other modules in INS. An LSTM module is a recurrent network module capable of storing values for both short and long periods of time	1. Better vision of complex dependencies than recurrent models 2. Networks are less sensitive to data outliers	1. Long-term dependencies are used with low quality 2. It is difficult to parallelize calculations 3. Longer time to train

Most studies with the use of AI for the analysis of microneurosurgical performance were conducted on models of the simplest surgical procedures, separate elementary phases of operations (for example, suturing, making incisions). Certainly, pilot studies in this area typically start with simplified models. However, surgery is a complex set of various factors that affect the surgical technique and the results of manipulations which are difficult to take into account during an experiment. And, therefore, the transfer of machine learning models from experimental conditions to real practice cannot ensure high quality-work, thus reducing their value.

## Conclusion

Despite the rapid development of machine learning methods in the field of clinical medicine, they are in the initial phase of approbation in the tasks of evaluating microsurgical techniques so far, and they do not seem to be introduced into everyday clinical practice in the nearest future. However, there are all grounds to believe that the use of machine learning technologies, computer vision in particular, in microsurgery has a good potential to improve the process of learning microsurgical techniques. And this serves a good prerequisite for the development of a special area of artificial intelligence in the field of microneurosurgery.
